# Mapping QTLs and association of differentially expressed gene transcripts for multiple agronomic traits under different nitrogen levels in sorghum

**DOI:** 10.1186/s12870-015-0696-x

**Published:** 2016-01-13

**Authors:** Malleswari Gelli, Sharon E. Mitchell, Kan Liu, Thomas E. Clemente, Donald P. Weeks, Chi Zhang, David R. Holding, Ismail M. Dweikat

**Affiliations:** Department of Agronomy and Horticulture, University of Nebraska, Lincoln, NE 68583 USA; Department of Biochemistry, University of Nebraska, Lincoln, NE 68588 USA; Center for Plant Science Innovation, University of Nebraska, Lincoln, NE 68588 USA; School of Biological Sciences, University of Nebraska, Lincoln, NE 68588 USA; Institute of Genomic Diversity, Cornell University, Ithaca, NY 14853 USA

**Keywords:** Sorghum, Agronomic traits, Differentially expressed gene transcripts, Genotyping-by-sequencing, Nitrogen fertilizer, QTL mapping, Illumina RNA-seq

## Abstract

**Background:**

Sorghum is an important C_4_ crop which relies on applied Nitrogen fertilizers (N) for optimal yields, of which substantial amounts are lost into the atmosphere. Understanding the genetic variation of sorghum in response to limited nitrogen supply is important for elucidating the underlying genetic mechanisms of nitrogen utilization.

**Results:**

A bi-parental mapping population consisting of 131 recombinant inbred lines (RILs) was used to map quantitative trait loci (QTLs) influencing different agronomic traits evaluated under normal N (100 kg.ha^−1^ fertilizer) and low N (0 kg.ha^−1^ fertilizer) conditions. A linkage map spanning 1614 cM was developed using 642 polymorphic single nucleotide polymorphisms (SNPs) detected in the population using Genotyping-By-Sequencing (GBS) technology. Composite interval mapping detected a total of 38 QTLs for 11 agronomic traits tested under different nitrogen levels. The phenotypic variation explained by individual QTL ranged from 6.2 to 50.8 %. Illumina RNA sequencing data generated on seedling root tissues revealed 726 differentially expressed gene (DEG) transcripts between parents, of which 108 were mapped close to the QTL regions.

**Conclusions:**

Co-localized regions affecting multiple traits were detected on chromosomes 1, 5, 6, 7 and 9. These potentially pleiotropic regions were coincident with the genomic regions of cloned QTLs, including genes associated with flowering time, *Ma3* on chromosome 1 and *Ma1* on chromosome 6, gene associated with plant height, *Dw2* on chromosome 6. In these regions, RNA sequencing data showed differential expression of transcripts related to nitrogen metabolism (Ferredoxin-nitrate reductase), glycolysis (Phosphofructo-2-kinase), seed storage proteins, plant hormone metabolism and membrane transport. The differentially expressed transcripts underlying the pleiotropic QTL regions could be potential targets for improving sorghum performance under limited N fertilizer through marker assisted selection.

**Electronic supplementary material:**

The online version of this article (doi:10.1186/s12870-015-0696-x) contains supplementary material, which is available to authorized users.

## Background

Sorghum (*Sorghum bicolor* (L.) Moench) is the fifth most cultivated cereal crop worldwide (http://www.fao.org/3/a-ax443e.pdf) and also an important source of fodder, fiber and biofuel [1]. Sorghum performs C_4_ photosynthesis like maize and sugarcane, and uses Nitrogen, CO_2_ and water more efficiently than maize and most C_3_ plants [2]. Sorghum is an important model for genome analysis among the C_4_ grasses because its genome is relatively small (~818 Mbp) [3], and the cultivated species is diploid (2n = 20). Due to its deep root system, sorghum is drought tolerant and is preferentially grown in water-limited environments [4]. Despite being a C_4_ crop, sorghum still relies on applied fertilizer to achieve maximal yields. Nitrogen (N) is the macronutrient which is often limiting sorghum production. N is the most abundantly absorbed mineral nutrient by plant roots [5] and 75 % of the leaf N is allocated to the chloroplasts [6]. As nitrogen is an essential part of many biomolecules, it comprises 1.5 to 2 % of plant dry matter and 16 % of the total plant protein [7].

N fertilizer application is expected to rise approximately three-fold in the next 40 years [8]. In general, plants absorb less than half of the applied fertilizer [7]. Both phosphorus and potassium are immobile nutrients in the soil and are generally not vulnerable for leaching. However, nitrogen is a mobile nutrient and when present in excess, it is released in to the atmosphere through volatilization or lost through leaching and ground water runoff, of which both have adverse environmental effects [8]. Excess N fertilizer application is a major economic cost to farmers, and also leads to acidification of soils [9]. Because of their potential positive effects on improving economic returns and limiting global climate change, lowering fertilizer input and breeding plants with better nitrogen use efficiency (NUE) are two major goals of research in plant nutrition [10]. As a function of multiple interacting genetic and environmental factors, the molecular basis of NUE is complex. NUE is defined as the grain yield [11] or fresh/dry matter produced [8] per unit of available N in the soil. Uptake of N from the soil involves a variety of transporters, and a number of enzymes for assimilation and transfer of the absorbed N into amino acids and other compounds [12]. However, little is known about how these processes are regulated especially under different N conditions.

QTL analysis, based on high density linkage maps, is a powerful tool for dissecting the genetic basis underlying complex traits [13]. QTL mapping studies have been conducted under different N conditions for NUE and other agronomic traits in maize [14], *Arabidopsis* [15], and rice [16, 17]. QTLs associated with low-nitrogen tolerance were detected in rice [18] and barley [19] for different traits, at the seedling stage. In barley, Mickelson et al. [20] mapped a QTL for grain protein concentration, which is homologous to a durum wheat grain protein QTL mapped by Joppa et al. [21]. QTLs for NUE and enzymes involved in nitrogen metabolism were reported in wheat [22] and QTLs for glutamine synthetase (GS) activity were co-localized with those for grain N [23] and confirmed in another population [24]. In wheat, Quraishi et al. [25] identified 11 major regions controlling NUE, which co-localized with key developmental genes such as *Ppd* (photoperiod sensitivity), *Vrn* (vernalization) and *Rht* (reduced height). However, there are no previous QTL mapping reports for agronomic traits tested under different nitrogen levels in sorghum. Significant genotypic differences for N utilization efficiency have been documented in sorghum [26, 27]. N utilization of genotypes varied with different nitrogen sources, nitrogen amounts and other environmental conditions [28]. Thus, there is good reason to believe that improvements in N utilization efficiency in sorghum can be achieved using genetic approaches.

Different kinds of DNA based low-throughput marker systems such as restriction fragment length polymorphism (RFLP), amplified fragment length polymorphism (AFLP), and simple sequence repeat (SSR) markers have been developed and used to investigate the variants and quantitative trait loci (QTLs) controlling >150 traits in sorghum. AFLPs, SSRs and RFLPs were used for generating the dense linkage maps [29]. Diversity Array Technology was evolved [30] as a cost effective hybridization-based alternative to the gel-based marker technologies, which offers a multiplexed genotyping independent of sequence information. DArT markers were developed for sorghum and used for genotyping a diverse set of sorghum lines and a bi-parental mapping population [31]. With the availability of sorghum whole genome sequence [32], Mace et al. [4] generated a single, reference consensus map by integrating six independent sorghum genetic maps containing 2029 unique loci consists of SSRs, AFLPs, and DArT markers. Using this as a framework map, Mace and Jordon et al. [33] mapped 35 major effect genes commonly observed in segregating mapping populations onto a common reference map to enable sorghum researchers link the information of QTLs and select the major genes. Furthermore, Mace et al. [34] projected 771 QTL relating to 161 unique traits from 44 studies onto the sorghum consensus map, which is useful for development of efficient marker-assisted breeding strategies. With the advent of high-throughput DNA sequencing technologies, it became possible to re-sequence genomes and detect single nucleotide polymorphisms (SNPs) which can be used for rapid genotyping [35]. Zou et al. [36] developed a linkage map based on SNPs generated from whole-genome re-sequencing by the Illumina Genome Analyzer IIx as described by Huang et al. [37] and used it for detecting QTLs for important agronomic traits under contrasting photoperiods in sorghum. However, it remains costly to employ whole-genome sequencing to evaluate multiple individuals in mapping populations. Next generation sequencing of a reduced representation genomic library, where fewer sequence reads are needed to obtain meaningful information compared to whole genome sequencing, is a convenient approach for capturing genetic variation. Genotyping-by-sequencing (GBS) is an efficient strategy for constructing multiplexed reduced representation library [38]. This technique has successfully been applied to generate high-density genetic maps and QTL mapping in several plant species [39].

In this study, we used SNPs generated from GBS technology to develop a linkage map and which then used to map QTLs for different agronomic traits in RIL population of sorghum. This process of QTL detection enabled us to link variation at the trait level to the variation at sequence level. However, a QTL may contain tens to hundreds of genes, figuring out the genes responsible for trait variation is a major challenge. With the advancement of sequencing technology, transcriptome comparisons were made between different sorghum genotypes at different tissue levels and at different growing conditions [40–44]. In addition, Morokoshi et al. [44] compiled all these datasets and developed a transcriptome database for sorghum which will be useful to researchers for transcriptome comparisons. The desire to identify the underlying genes responsible for trait variation in QTL regions has been increasing and to this end, we used previously generated high throughput Illumina-based RNA sequencing data [43] to identify differentially expressed gene transcripts in QTL regions. By further evaluation, the resulting candidate genes could be potential targets for improving N-stress tolerance and nitrogen utilization of sorghum and related crops.

## Methods

### Plant material

A mapping population derived from a cross between the inbred lines CK60 and China17 was used in this study. CK60, a public sorghum line, which is short, photoperiod-sensitive, late-maturing U.S. sorghum line and an inefficient N user. China17, a photoperiod-insensitive Chinese sorghum line was provided by Dr. Jerry Maranville (University of Nebraska, Lincoln, USA), uses nitrogen more efficiently than CK60 and has higher assimilation efficiency indices at both low and high soil nitrogen levels [45]. China17 retains higher phosphoenolpyruvate carboxylase (PEPcase) activity than CK60 when grown under low N conditions [45]. The seedlings of China17 had greater root and shoot mass than CK60 under both low N and normal N conditions [43]. Each of the 131 RILs was derived from a single F_2_ plant following a single seed descent method until the F_7_ generation.

### Experimental design

The F_7_ RILs and the two parents (CK60 and China17) were evaluated in an alpha lattice incomplete block design under two N levels with two independent replicates each for two years (2011 and 2012). The two N treatments were low N (LN, 0 kg.ha^−1^ fertilizer) and normal N (NN, 100 kg.ha^−1^ anhydrous ammonia fertilizer). The preceding crops were soybean in the NN field and oats or maize in the LN filed. The LN field had not received nitrogen fertilizer since 1986. The soil testing was done by collecting soil samples from 0 to 12 in. and 12–24 in. randomly across the NN and LN fields and results were described in Additional file 1. Single-row plots measuring five meters long at 0.75 m row spacing were sown at a density of 50 seeds for each RIL and parents. All entries were planted on the same day in conventionally tilled plots and maintained under rain fed conditions.

### Phenotyping of important agronomic traits

Three plants were randomly selected for each genotype for phenotypic evaluation of eleven agronomic traits. The measured phenotypes include leaf chlorophyll content at three different stages of plant growth: before flowering (vegetative stage, Chl1), during flowering (Chl2) and at maturity (Chl3); plant height (PH, from base of the plant to tip of the head, in centimeters); and days to anthesis (AD, no. of days from planting to 50 % anthesis). Stover moisture contents (MC1) and head moisture contents (MC2) were calculated as the percent difference between wet and dry weights. Total biomass yield (BY, t.ha^−1^), grain yield (GY, t.ha^−1^), 1000 seed weight in grams (Test weight, TW) and grain-to-stover ratio (GS, %) were calculated and recorded from NN and LN fields. Haussmann et al. [46] described that the upper six leaves are a good source for measuring the greenness of leaves since they are photosynthetically active at anthesis and contribute nutrients to the grain [47]. In this study, chlorophyll contents were measured in the 3^rd^ leaf from the top using a portable chlorophyll meter model SPAD-502 (Minolta, Japan). In summary, the phenotypes were classified into three groups, chlorophyll contents (Chl1, Chl2, and Chl3), morphological traits (PH, AD, MC1, and MC2), and yield-related traits (BY, GY, TW and GS).

### Statistical analysis

The statistical model adopted for the alpha lattice incomplete block design in each N condition was *Y*_*ijk*_ = μ + *g*_*i*_ + *r*_*j*_ + *b*_k(j)_ + e_ij_. *Y*_*ijk*_ is the response of i^th^ genotype in k^th^ bock of j^th^ replication, μ is the grand mean, *g*_i_ is the genotype or line effect, r_j_ is the replication effect, *b*_k(j)_ is the random block k (k = 1…n) effect within replicate with b_k(j)_ ~ N(0, σ^2^_b_) and e_ij_ is the residual term with ~ N(0, σ^2^_e_). Analysis of variance (ANOVA) for eleven traits was performed for each individual environment using the PROC MIXED procedure [48] of SAS version 9.2 (SAS Institute, 2008) where the genotype was considered as fixed, replications and blocks as random effects. The phenotypic data, from both seasons (2011 and 2012), were pooled to obtain single trait values for each family under NN and LN [13]. ANOVA was performed on pooled data by considering that genotype effect is fixed and environments (years), replication within environments, blocks within environments, and genotype by environment (GxE) interaction effects are random. Narrow-sense heritability with standard error was estimated using the PROC MIXED procedure of SAS version 9.2. For the heritability estimates, parental lines data were excluded, and estimates followed a method described by Holland et al. [49]. Pearson’s correlation coefficients between traits were calculated for the least square genotype means using the PROC CORR procedure of SAS. The RIL trait data were subjected to normality test using PROC UNIVARIATE to determine its suitability for QTL analysis.

### High-throughput Genotyping and Linkage map construction

Total genomic DNA of the RILs and their parents were isolated from leaf tissues using a DNeasy Plant Mini Kit (Qiagen). DNA (500 ng) from each sample was digested with ApeKI (New England Bio-labs, Ipswich, MA), a type II restriction endonuclease that recognizes a degenerate 5 bp sequence (5’-GCWGC) and creates 5’ overhangs. Adapters with specific barcodes [38] were then ligated to the overhanging sequences using T_4_ ligase. A set of 96 DNA samples, each sample with a different barcode adapter, were combined and purified (Quick PCR Purification Kit; Qiagen, Valencia, CA) according to the manufacturer’s instructions. DNA fragments containing ligated adapters were amplified with primers containing complementary sequences for each adapter. PCR products were then purified and diluted for sequencing [38]. Single-end, 100 bp reads were collected for one 48- or 96-plex library per flow cell channel on a Genome Analyzer IIx (GAIIx; Illumina, Inc., San Diego, CA) [50] at Cornell University, USA.

Raw reads obtained from GAIIx were filtered [38] and aligned to the sorghum reference genome version 1.4 [32]. The genotypes of the population were determined based on the procedure described by Elshire et al. [38]. The biallelic SNP markers were checked for polymorphism between the parents. Prior to map construction, all polymorphic SNPs were checked by the chi-square (*χ*2) test for the goodness of fit against a 1:1 segregation ratio at the 0.05 probability level. SNPs with >70 % missing data were removed from data set. A total of 668 SNPs were selected and used for constructing linkage maps using Mapmaker/EXP 3.0 along with IciMapping (Inclusive composite interval mapping) V3.2 [51]. The genetic distance (cM) was calculated using the Kosambi mapping function.

### QTL analysis

The composite interval mapping method of WinQTLcart2.5 [52] was used for QTL detection. QTL analysis was performed based on averaged mean values of each trait across two NN and two LN environments respectively. The walking speed chosen for all traits was 1 cM. Cofactors were determined using the forward and backward step-wise regression method with a probability in and out of 0.1 and a window size of 10 cM. A thousand-permutation test was applied to each data set to decide the LOD (logarithm of odds) thresholds (*P* ≤ 0.05) to determine significance of identified QTLs [53]. A 2-LOD support interval was calculated for each QTL to obtain a 95 % confidence interval. Adjacent QTLs on the same chromosome for the same trait were considered different when the support intervals were non-overlapping. The contribution rate (R^2^) was estimated as the percentage of variance explained by each QTL in proportion to the total phenotypic variance. The additive effect of a putative QTL was estimated by half the difference between two homozygous classes. QTLs were named according to McCouch et al. [54] and alphabetical order was used for QTLs on the same chromosome. QTLs with a positive or negative additive effect for a trait imply that the increase in the phenotypic value of the trait is contributed by alleles from CK60 or China17.

### Detection of differentially expressed gene transcripts in the QTL intervals

In an earlier study [43], we detected several common DEG transcripts between the transcriptomes of seven sorghum genotypes (four low-N tolerant and three low-N sensitive) using Illumina RNA sequencing. Transcriptomes were prepared from root tissues of 3 week old seedlings grown under N-stress from four N-stress tolerant (China17, San Chi San, KS78 and high NUE bulk) and three sensitive (CK60, BTx623 and low NUE bulk) genotypes. In the present study, we used the RNA-seq data generated earlier in order to check the differential expression of gene transcripts between CK60 and China17 in the QTL regions. Pair-wise comparison was made between the transcriptomes of CK60 and China17 to detect DEG transcripts. The cutoff of log_2_-fold value >1 (2-fold absolute value) and adjusted *P*-value <0.001 (FDR) were used for determining significant DEG transcripts.

## Results

### Statistical analysis of phenotypic data

Mean values of 11 traits measured for parents (CK60, and China17) and the RIL population under NN and LN environments are given in Tables 1 and 2, respectively. The mean chlorophyll content was higher at flowering than at vegetative and mature stages under both N-conditions. CK60 retained more chlorophyll at all stages compared to China17 and the mean chlorophyll content of the RIL population was lower under LN compared to NN conditions. The plant height of CK60 was reduced by 23 cm, while that of China17 remained the same under LN compared to NN. Days to anthesis for the two parental lines were also significantly affected by N-condition, and LN delayed flowering in both parents. Compared to China17, the flowering was delayed more in CK60 under both N-levels. The biomass yield of CK60 was lower than China17 in both N conditions. The grain yield was also significantly different between the two parents; CK60 had lower grain yield under the two N-conditions. The average values of biomass and grain yield for the RILs were greatly reduced from NN to LN conditions, respectively. Similarly, the test weight of China17 was higher than CK60 under both N-conditions. The grain/stover ratio of China17 was decreased almost half, while no significant change was observed for CK60 under LN compared to NN. In contrast, the stover and head moisture contents of CK60 were higher than China17 under both N-conditions. The average of grain/stover ratio and stover moisture contents of the RILs remained the same under both N conditions but the average of head moisture content in the RIL population was increased under LN conditions.

**Table 1:** Descriptive statistics, *h* ^*2*^, and mean squares of ANOVA results for the traits measured across two normal-N conditions in CK60 x China17 RIL population

Category	Source of variation	Df	Chl1	Chl2	Chl3	PH	AD	MC1	MC2	BY	GY	TW	GS
Descriptive statistics	CK60		49.8	55.6	53.6	99	71.5	68.6	24.8	7.69	2.89	20.3	0.52
	China17		46.6	52.7	48.3	150	66.3	65	19.5	14.6	6.25	31.6	0.95
	RIL Mean		47.8	53.3	47.2	161.3	67	66.5	19.4	11.2	3.39	23.6	0.47
	Std		4.09	3.72	5.85	35	4.2	3.32	6.34	3.99	1.49	3.15	0.16
	Min		38.7	38.2	32.3	70	55.1	52.8	8.16	3.09	0.4	14.4	0.05
	Max		58.4	62.2	62.5	236.5	85.9	76.1	46.8	24.2	9.04	29.9	0.88
	h^2^ (%)		71	56	51	64	61	40	53	62	55	64	39
	SE (%)		6	9	10	7	8	12	9	0.8	9	7	12
ANOVA	Env	1	626.9	4276***	17016***	40478	11333***	347.6	4500	271.6	32.8*	946.9	0.07
	Rep(Env)	2	89.1*	9.93	57.8	10976**	55.6*	191.1**	2501***	34.6	2.57	1483***	0.02
	Blk(Env*Rep)	44	12.5	12.0*	40.4***	429*	11.3	14.8	31.7	13.1	2.43*	5.69	0.02
	Line	130	50.9***	41.9***	105.3***	4037***	49.6***	34.3**	123.9***	49.4***	6.8***	28.0***	0.08**
	Env*Line	104	15.6**	18.1***	58.2***	1634***	20.5**	21.8**	59.3***	19.6***	3.2***	10.5***	0.048***
	Residual	190	9.74	8.06	16.7	290	12.8	12.7	26.7	10.5	1.54	5.2	0.02

Df, degrees of freedom; chlorophyll contents at vegetative stage (Chl1), at anthesis (Chl2), and at maturity (Chl3); PH, plant height (cm)

AD, days to anthesis; MC1, % stover moisture content; MC2, % head moisture content; BY, biomass yield (t.ha^−1^); GY, grain yield (t.ha^−1^)

TW, test weight (g); GS, grain/stover ratio (%). Std, standard deviation; h^2^ (%), narrow sense heritability; SE (%), standard error %; ***P < 0.0001; **P < 0.01; *P < 0.05

**Table 2:** Descriptive statistics, h^2^, and mean squares of ANOVA results for the traits measured across two low-N conditions in CK60 x China17 RIL population

Category	Source of variation	Df	Chl1	Chl2	Chl3	PH	AD	MC1	MC2	BY	GY	TW	GS
Descriptive statistics	CK60		31.8	39.5	40.2	76.3	90.5	68.6	34.4	3.75	1.21	20.2	0.49
	China17		32.7	33.9	28.7	153.1	77.1	60.7	23.8	6.83	2.72	28.3	0.46
	RIL Mean		33.3	36.8	31.8	131.7	82.6	66.2	27.4	6.43	1.86	20.3	0.42
	Std Dev		3	3.9	5.4	38.7	7.8	3.2	9	2.1	0.79	3.3	0.14
	Min		27.3	25.2	12.3	55.9	66.7	55.5	13.4	2.91	0.06	12.1	0.01
	Max		40.2	48.1	46.2	214	108.2	74.6	57.8	13.2	5.02	27.8	0.96
	h^2^ (%)		59	43	50	80	75	71.6	76	48	47	75	32
	SE (%)		8	12	10	4	5	6	5	10	10	5	14
ANOVA	Env	1	360.5	16104***	24768**	4740.9	54521***	186	264	435.9*	163***	368.1	6.32*
	Rep(Env)	2	87.7*	23.3	131.7*	771.4	48.85	129***	670.5**	21.4	0.16	1779***	0.22**
	Blk(Env*Rep)	44	16.6*	19.1	18.66	412.3**	36.67	7.8	35	5.9	0.69	6.14	0.01
	Line	130	27.2***	45.6**	87.7**	4475***	167.4***	31.7***	238.0***	15.0**	1.97**	32.7***	0.05*
	Env*Line	104	12.1	27.3***	44.03***	1001***	46.8*	9.9***	66.2***	8.87**	1.17**	9.48**	0.03***
	Residual	190	10.7	13.9	15.13	189.9	31.78	6.58	34.2	5.5	0.75	5.67	0.02

Df, degrees of freedom; chlorophyll contents at vegetative stage (Chl1), at anthesis (Chl2), and at maturity (Chl3); PH, plant height (cm); AD, days to anthesis; MC1, % stover moisture content; MC2, % head moisture content; BY, biomass yield (t.ha^−1^); GY, grain yield (t.ha^−1^); TW, test weight (g); GS, grain/stover ratio (%). Std, standard deviation; h^2^ (%), narrow sense heritability; SE (%), standard error %; ***P < 0.0001; **P < 0.01; *P < 0.05

The narrow sense heritability (*h*^*2*^) was estimated for each trait measured under both N conditions (Tables 1 and 2). Under NN, the heritability estimates of the 11 traits ranged from 39 to 71 %. Chlorophyll at the vegetative stage had the highest *h*^*2*^ value followed by plant height and test weight. Grain/stover ratio had the lowest heritability estimate. Under LN, *h*^*2*^ values ranged from 32 to 80 %. Plant height had the highest *h*^*2*^ values and grain/stover ratio had the lowest *h*^*2*^ value. ANOVA showed significant phenotypic variation for all the traits among RILs (Tables 1 and 2). GxE interaction was mainly associated with differences in magnitude of effects between years. Therefore, phenotypic data from 2011 and 2012 seasons were averaged separately for NN and LN conditions. GxE interactions were significant for all the traits except chlorophyll at the vegetative stage across two LN environments. Genotype variance was greater than GxE interaction variance for all traits across NN and LN environments (Tables 1 and 2).

### Correlation of the traits

The focus of this work was evaluation of the genetic control of traits under NN and LN conditions in sorghum. Correlation coefficients based on the line means among three chlorophyll contents, yield-related traits and other morphological traits showed that most of the traits tested under the contrasting N conditions were significantly correlated (*P* < 0.05) (Table 3). Interestingly, leaf chlorophyll contents measured at three different stages of plant growth were negatively correlated with most of the yield-related (biomass yield, grain yield and test weight) and morphological traits (plant height, days to anthesis and head moisture content) in both N-conditions (Table 3). Under NN conditions, significant positive correlations were observed between chlorophylls and stover moisture content (*P* < 0.01). In addition, plant height had significant positive correlation with biomass and grain yield in both N conditions. Highest positive correlation was observed between biomass and grain yield in both NN and LN environments. Days to anthesis was positively correlated with stover and head moisture contents under both N conditions. Grain/stover ratio was not significantly correlated with many traits, but it had significant positive correlation with grain yield.

**Table 3:** Correlation coefficient of the traits investigated

	Chl1	Chl2	Chl3	PH	AD	MC1	MC2	BY	GY	GS	TW
Chl1		0.76***	0.65***	−0.35***	−0.17*	0.134	−0.065	−0.069	0.066	0.144	−0.065
Chl2	0.77***		0.73***	−0.38***	−0.36***	0.08	−0.23**	−0.18*	0.085	0.30**	−0.016
Chl3	0.57***	0.58***		−0.51***	0.077	0.22*	0.163	−0.162	−0.016	0.118	−0.24**
PH	−0.62***	−0.51***	−0.52***		−0.16	−0.22*	−0.40***	0.54***	0.39***	0.022	0.43***
AD	−0.26**	−0.30**	0.15	0.13		0.34***	0.76***	0.003	−0.19*	−0.28**	−0.47***
MC1	0.28**	0.16	0.38***	−0.40***	0.19*		0.39***	−0.046	−0.097	0.037	−0.21*
MC2	−0.003	−0.21*	0.27**	−0.078	0.51***	0.38***		−0.04	−0.35***	−0.45***	−0.49***
BY	−0.64***	−0.56***	−0.37***	0.63***	0.25**	−0.24**	0.12		0.75***	0.033	0.38***
GY	−0.56***	−0.36***	−0.35***	0.50***	0.142	−0.31**	−0.27**	0.73***		0.60***	0.40***
GS	−0.078	0.04	−0.1	0.043	−0.132	−0.151	−0.53***	−0.094	0.52***		0.26**
TW	−0.23**	0.02	−0.166	0.20*	−0.1	−0.24**	−0.21*	0.23**	0.31**	0.064	

The numbers below the diagonal are correlation coefficients under normal N environments and numbers above the is diagonal are correlation coefficients under low N environments. Chlorophyll contents at vegetative stage (Chl1), at anthesis (Chl2), and at maturity (Chl3); PH, plant height (cm); AD, days to anthesis; MC1, % stover moisture content; MC2, % head moisture content; BY, biomass yield (t.ha^−1^); GY, grain yield (t.ha^−1^); TW, test weight (g); GS, grain/stover ratio (%). ***P < 0.0001; **P < 0.01; *P < 0.05

### Linkage mapping and QTL analysis

**Fig. 1:**
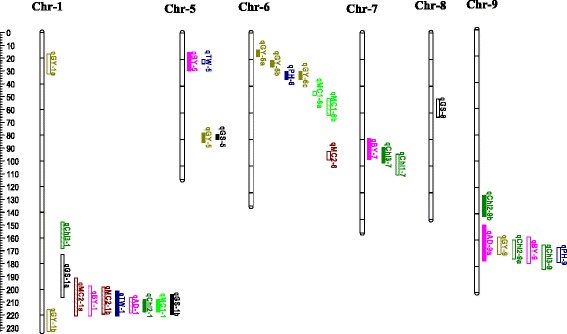
QTLs mapped to the linkage groups for 11 agronomically important traits across two normal N and two low-N conditions. Chr, indicate chromosome. Chlorophyll contents at vegetative stage (Chl1), at anthesis (Chl2), and at maturity (Chl3); plant height (PH, cm), days to anthesis (AD, days), stover moisture content (MC1,%), head moisture content (MC2,,%), biomass yield (BY, t.ha^−1^), grain yield (GY, t.ha^−1^), test weight (TW, g), and grain/stover ratio (GS, %); each trait was shown with different color; open bars indicates QTLs detected under NN, closed bars indicates QTLs detected under LN and open bar with strikes indicates QTLs detected consistently across environments. Supported intervals for each QTL are indicated by the length of vertical bars. Chr doesn’t contain QTLs not shown here. Left side scale is in cM

Polymorphic SNP markers between CK60 and China17 were identified by the GBS pipeline. A linkage map was developed with 642 polymorphic SNPs (Additional file 2) with an average inter marker distance of 2.55 cM. The resulting linkage map comprised of 10 linkage groups and map spanning a total length of 1641 cM. Composite interval mapping detected a total of 38 QTLs for 11 traits analyzed across NN and LN environments. No significant QTLs were detected on chromosomes 2, 3, 4 and 10 (not shown in Fig. 1). The number of QTLs per trait ranged from one to four, and is listed in Tables 4 and 5 and shown in Fig. 1. Across two NN conditions, four QTLs for chlorophyll contents were detected including one QTL each for chlorophyll at vegetative and flowering stage, and two QTLs for chlorophyll at maturity explaining phenotypic variation range from 7.1 to 50.8 % (Table 4). Six QTLs were identified for four morphological traits including one major QTL for days to anthesis on chromosome 1, for which the CK60 allele delayed flowering by 3.6 days. Two QTLs each for stover and head moisture contents were detected under NN conditions. For all these QTLs, the CK60 allele contributed to increase the chlorophyll contents and the moisture contents. In contrast, the China17 allele contributed to an increase in the plant height by 39.8 cm for the QTL detected on chromosome 9. Similarly, we detected eight significant QTLs for yield-related traits. Of the eight detected, two QTLs are for biomass yield, three for grain yield, one for test weight and two for grain/stover ratio. For the two QTLs detected for biomass yield, China17 allele increased the biomass yield by 1.8 t.ha^−1^. For grain yield, CK60 allele increased grain yield by 0.5 t.ha^−1^ for the two QTLs on chromosome 1 and China17 allele increased grain yield for the other QTL on chromosome 9. CK60 allele responsible for an increase in the test weight of seeds for the major QTL detected on chromosome 5 for test weight. In contrast, the China17 allele increased the grain/stover ratio for two QTLs.

**Table 4:** QTLs detected for 11 traits using the SNP linkage map across two normal N conditions

Trait	QTL	Chr	Position (cM)	Flanking marker	Interval (cM)^a^	LOD score	Additive^b^	R^2^ (%)^c^
Chl-1	q*Chl1-7*	7	97.8	S7_60490830 - S7_60947414	91.1–106.6	2.76	1.18	7.1
Chl-2	q*Chl2-9a*	9	167.4	S9_45363122 - S9_58417131	159.9–174.4	10.2	3.20	50.8
Chl-3	qChl3-1	1	157.6	S1_50614823-S1_50837764	147.6–168.1	3.72	2.77	8.10
	q*Chl3-9*	9	169.4	S9_45363122 - S9_58417132	163.6–182.6	2.64	2.13	11.8
PH	q*PH-9*	9	171.4	S9_45363122 - S9_58417133	165.8–177.2	4.33	−39.8	44.2
AD	qAD-1	1	213.9	S1_61836509 – S1_62490042	208.7–218.3	5.15	3.6	16
MC1	q*MC1-6a*	6	45.6	S6_48858797 - S6_49609588	44–46.8	13.5	2.39	29.1
	q*MC1-6b*	6	54.6	S6_52982294 - S6_54274803	49.3–61.9	4.84	1.47	16.2
MC2	q*MC2-1*	1	203	S1_55726325 - S1_57821154	191.3–210.9	4.57	2.70	15.3
	q*MC2-6*	6	91.6	S6_57001245 - S6_57540748	88.8–95.5	3.19	1.96	8.56
BY	q*BY-1*	1	204	S1_55726325 - S1_57821154	197.3–220.9	2.60	−1.82	10.8
	q*BY-9*	9	167.4	S9_45363122 - S9_58417134	157.6–177.7	5.34	−2.41	33.8
GY	q*GY-1a*	1	28.3	S1_2983876 - S1_3356469	16.7–32.3	3.84	0.52	9.84
	q*GY-1b*	1	223	S1_64266923 - S1_71768492	215.9–232.7	3.59	0.92	17.3
	q*GY-9*	9	161.4	S9_45363122 - S9_58417134	157.6–170.9	4.82	−0.67	17.6
TW	qTW-5	5	22	S5_44956096 - S5_45759643	20–23.1	4.51	1.72	15.0
GS	q*GS-1a*	1	190	S1_54743129 - S1_54776428	173.1–206.4	3.30	−0.06	11.3
	q*GS-8*	8	56.5	S8_398073 - S8_5494183	49.6–63.3	3.84	−0.09	17.6

Chlorophyll contents at vegetative stage (Chl1), at anthesis (Chl2), and at maturity (Chl3); PH, plant height (cm)

AD, days to anthesis; MC1, % stover moisture content; MC2, % head moisture content; BY, biomass yield (t.ha^−1^)

GY, grain yield (t.ha^−1^); TW, test weight (g); GS, grain/stover ratio (%). ^a^2.0-LOD drop support interval of the QTL; ^b^Additive effect: positive values of the additive effect indicate that alleles from CK60 were in the direction of increasing the trait score and vice versa; ^c^ Percentage of phenotypic variation explained by the QTL. The SNP underlined is the corresponding SNP of QTL

**Table 5:** QTLs detected for 11 traits using the SNP linkage map across two low-N conditions

Trait	QTL	Chr	Position (cM)	Flanking marker	Interval (cM)^a^	LOD score	Additive^b^	R^2^ (%)^c^
Chl-2	q*Chl2-1*	1	211	S1_61786623 - S1_61836509	208.1–217.7	3.28	−1.26	8.69
	q*Chl2-9b*	9	136	S9_55230722 - S9_56646280	126.1–142.2	3.07	1.25	8.49
Chl-3	q*Chl3-1*	1	157.8	S1_50614823 - S1_50837764	150.6–178.1	3.29	1.79	10.1
	q*Chl3-7*	7	87.7	S7_57772979 - S7_60426792	85.9–97.8	4.57	2.71	14.2
PH	q*PH-6*	6	30.8	S6_41970042 - S6_43222258	28.9–34.8	5.30	−16.4	13.2
AD	qAD-1	1	210.8	S1_61709596- S1_61786623	206.5–218.1	5.15	3.63	16.7
	q*AD-9a*	9	159.4	S9_45363122 - S9_58417131	148.8–176	3.29	−3.00	16.4
MC1	q*MC1-1*	1	211	S1_61786623 - S1_61836509	208.1–217.7	3.14	1.44	10.0
	q*MC1-6b*	6	54.6	S6_52982294 - S6_54274803	49.3–61.9	2.69	1.14	6.97
MC2	q*MC2-1a*	1	203	S1_55726325 - S1_57821154	201.3–220.9	4.74	4.74	16.4
	q*MC2-1b*	1	209.4	S1_59080688- S1_59595161	198.2–219.4	8.10	4.44	20.3
BY	q*BY-5*	5	20	S5_44956096 - S5_45759643	14.5–28.4	2.95	0.72	9.04
	*qBY-7*	7	85.9	S7_57557894 - S7_57772979	79.3–95.1	3.86	−1.01	12.5
GY	q*GY-5*	5	78.6	S5_59373257 - S5_59373316	75.3–82.2	3.22	0.37	10.8
	q*GY-6a*	6	15.8	S6_3799293 - S6_8141493	12.7–17.8	3.06	−0.25	9.83
	q*GY-6b*	6	22.3	S6_13884102 - S6_37768125	20.6–25.6	3.03	−0.38	11.0
	q*GY-6c*	6	30.8	S6_41970042 - S6_43222258	28.9–34.8	2.73	−0.23	8.00
TW	qTW-1	1	209.4	S1_59595161- S1_61709496	201.3–220.9	5.79	−1.79	17.9
GS	q*GS-1b*	1	212.7	S1_61786623 - S1_61836509	204.1–219.7	4.67	−0.08	14.4
	q*GS-5*	5	78.6	S5_59373257 - S5_59373316	76.2–80.1	5.30	0.07	14.6

Chlorophyll contents at vegetative stage (Chl1), at anthesis (Chl2), and at maturity (Chl3); PH, plant height (cm); AD, days to anthesis; MC1, % stover moisture content; MC2, % head moisture content; BY, biomass yield (t.ha^−1^); GY, grain yield (t.ha^−1^); TW, test weight (g); GS, grain/stover ratio (%). ^a^2.0-LOD drop support interval of the QTL; ^b^Additive effect: positive values of the additive effect indicate that alleles from Ck60 were in the direction of increasing the trait score and vice versa; ^c^ Percentage of phenotypic variation explained by the QTL. The SNP underlined is the corresponding SNP of QTL

Under LN conditions, 20 QTLs were found to be significant for 11 traits studied (Table 5, Fig. 1). We detected four QTLs for chlorophyll content including two each for chlorophyll at flowering and maturity. No significant QTLs were detected for chlorophyll content at the vegetative stage. For these QTLs, the China17 allele increased the chlorophyll content at flowering for the QTL on chromosome 1 and the CK60 alleles increased the chlorophyll contents for the other QTLs. We detected seven significant QTLs for morphological traits. One major QTL explaining 13.2 % of the phenotypic variation was associated with plant height with the allele from China17 increasing plant height by 16.4 cm. Two QTLs were detected for days to anthesis. The CK60 allele associated with the QTL on chromosome 1 delayed heading by 3.6 d, while the China17 allele, associated with the QTL on chromosome 9, delayed heading by 3 d. Two QTLs for stover moisture content and head moisture content were identified with presence of the CK60 alleles resulting in increasing the moisture contents. Nine significant QTLs were found for yield-related traits under LN conditions. Two QTLs were detected for biomass yield, of which the China17 allele contributed for increased biomass yield by 1.0 t.ha^−1^ for QTL on chromosome 5, while the CK60 allele increased biomass yield at other QTL. Four QTLs were identified for grain yield, of which the CK60 allele increased the grain yield for one QTL on chromosome 5 and China17 alleles improved the grain yield for all other QTLs. One significant QTL explaining 17.9 % of the phenotypic variation was detected for test weight on chromosome 1 with the China17 allele increasing test weight by 1.8 g. Two QTLs were found for grain/stover ratio on chromosomes 1 and 5. The China17 allele contributed to an increase the grain/stover ratio for QTL on chromosome 1 while the CK60 allele was responsible for increasing the grain/stover ratio at the other QTL on chromosome 5. The additive effect of a single QTL could explain 7 to 20.3 % of the total phenotypic variation.

### Differential expression of gene transcripts in the QTL regions

The previously generated Illumina RNA-sequencing data [43] was used to determine the variations in transcript abundance between nitrogen use inefficient (CK60) and efficient (China17) genotypes of sorghum. False discovery rate (FDR) ≤ 0.001 and the absolute value of |log_2_ -Ratio| ≥ 1 were used as thresholds to judge the significance of differences in transcript abundance of the same gene between two genotypes. Pair-wise comparison of the transcriptomes of CK60 and China17 seedling root tissues grown under N-stress revealed a total of 726 DEGs detected using v1.4 sorghum genome (Additional file 3). The sequences of all these DEGs compared to v2.1 sorghum genome and respective gene IDs were listed in Additional file 3. In addition, compared the sequences of polymorphic SNPs between CK60 and China17 to the sequences of DEG transcripts, and differential expression levels were listed in Additional file 2.

Out of 726 DGE transcripts observed between CK60 and China17 (Additional file 3), 108 DEGs were located in the vicinity of the QTL confidence intervals on chromosome 1, 6, 7, 8, and 9 (Additional file 3) and some of those were listed in Table 6. The QTL interval on chromosome 1 has 40 DEGs and chromosome 9 has 28 DEGs. Gene transcripts related to nitrogen metabolism (Ferredoxin-nitrate reductase), glycolysis (Phosphofructo-2-kinase), seed storage proteins, plant hormone metabolism (Gibberellin receptor GID1L2, Auxin response factor 2) were differentially expressed between CK60 and China17. The majority of these gene transcripts were expressed higher in CK60 than China17 under N-stress conditions in the seedling stage. For example, transcripts of Frigida, Auxin response factor 2 and translation elongation factor expressed six-fold higher in CK60 than China17. In contrast, magnesium transporter6, HSP21 and senescence associated protein were expressed higher in China17. A ferredoxin-nitrite reductase gene transcript which had higher expression in China17, coincided with the pleiotropic QTL region on chromosome 9.

**Table 6:** Differential expression of gene transcripts associated with QTLs detected using RNA-seq

Gene id (v1.4)	Chr	Start (bp)	logFC	Annoatation	Low N QTLs	Normal N QTLs
Sb01g032875	1	55828932	6.3	Translation elongation factor EF1B	qMC2-1a	qMC2-1, qBY-1
Sb01g032880	1	55840286	4	SPX domain-3	qMC2-1a	qMC2-1, qBY-1
Sb01g032920	1	55885605	6.84	Frigida putative expressed	qMC2-1a	qMC2-1, qBY-1
Sb01g033010	1	56047918	9.1	Retrotransposon protein,	qMC2-1a	qMC2-1, qBY-1
Sb01g033090	1	56202769	3.98	Mannose-binding lectin superfamily	qMC2-1a	qMC2-1, qBY-1
Sb01g033360	1	56595053	−5.2	Acetoacetyl-CoA thiolase 2	qMC2-1a	qMC2-1, qBY-1
Sb01g033410	1	56731990	4.27	Cation/carnitine transporter 3	qMC2-1a	qMC2-1, qBY-1
Sb01g033620	1	56944250	2.77	Metacaspase 1	qMC2-1a	qMC2-1, qBY-1
Sb01g034190	1	57636134	2.49	O-Glycosyl hydrolases	qMC2-1a	qMC2-1, qBY-1
Sb01g035910	1	59529076	9.28	Glutathione S-transferase	qMC2-1b	
Sb01g036330	1	59936853	−3.38	Ribosomal protein L16p/L10e family	qTW1	
Sb01g036790	1	60395728	2.56	Late embryogenesis abundant protein 1	qTW1	
Sb01g037480	1	61031332	4.1	Nicotianamine synthase 4	qTW1	
Sb01g038720	1	62214256	−7.69	LHT1 lysine histidine transporter 1		qAD-1
Sb01g041180	1	64497962	−3.32	HSP21 Heat shock protein 21		qGY-1b
Sb01g041350	1	64653444	2.63	Subtilisin-like serine protease 2		qGY-1b
Sb01g041390	1	64692370	−2.39	Senescence associated protein		qGY-1b
Sb01g041640	1	64933485	2.04	Oxidoreductase superfamily protein		qGY-1b
Sb01g041810	1	65045823	3.75	STRUBBELIG-RECEPTOR FAMILY 7		qGY-1b
Sb01g042500	1	65775142	1.99	Caleosin-related family protein		qGY-1b
Sb01g042530	1	65792345	2.89	MA3 domain-containing protein		qGY-1b
Sb01g044230	1	67360823	2.26	Polyamine oxidase 1		qGY-1b
Sb01g044810	1	67970813	3.04	MADS-box transcription factor family		qGY-1b
Sb01g045620	1	68676107	2.29	Lectin protein kinase family protein		qGY-1b
Sb01g047250	1	70350087	2.412	Leucine-rich repeat transmembrane protein kinase		qGY-1b
Sb01g047550	1	70645925	2.215	Tetratricopeptide repeat (TPR)-like superfamily protein		qGY-1b
Sb01g047650	1	70741548	−3.156	CCT motif family protein		qGY-1b
Sb01g047780	1	70919577	−8.348	Magnesium transporter 6		qGY-1b
Sb01g048000	1	71075448	1.861	Glutathione S-transferase		qGY-1b
Sb01g048030	1	71101357	3.126	Cytochrome P450, family 78,		qGY-1b
Sb01g048100	1	71159986	−4.194	LYM2 lysm domain GPI-anchored protein 2 precursor		qGY-1b
Sb01g048640	1	71596638	6.418	Leucine-rich repeat family protein		qGY-1b
Sb06g002090	6	3921155	3.276	F-box/RNI-like/FBD-like domains-containing protein	qGY-6a	
Sb06g002180	6	4089379	−2.511	UDP-glucosyltransferase	qGY-6a	
Sb06g003180	6	6636307	3.018	CAP (Cysteine-rich secretory proteins	qGY-6a	
Sb06g005420	6	13588101	6.889	expressed protein		
Sb06g006920	6	16373350	−2.546	purple acid phosphatase 27	qGY-6b	
Sb06g008990	6	26359064	2.375	Oxidoreductase, zinc-binding dehydrogenase family protein	qGY-6b	
Sb06g010870	6	30379011	3.616	Cytochrome P450, family 71	qGY-6b	
Sb06g011767	6	32144416	6.478	auxin response factor 2	qGY-6b	
Sb06g011770	6	32145882	6.844	C2H2-like zinc finger protein	qGY-6b	
Sb06g012040	6	32755618	2.708	Minichromosome maintenance (MCM2/3/5) family protein	qGY-6b	
Sb06g012280	6	33774679	2.815	UDP-Glycosyltransferase superfamily	qGY-6b	
Sb06g012290	6	33921889	4.708	Galactose oxidase/kelch repeat superfamily protein	qGY-6b	
Sb06g014250	6	39313831	4.128	multidrug resistance-associated protein 9	qGY-6b	
Sb06g014400	6	39867816	−3.085	HSP70 Heat shock protein 70		
Sb06g015520	6	43082617	3.501	B-block binding subunit of TFIIIC	qPH-6, qGY-6c	
Sb06g016160	6	44576681	2.202	seed storage 2S albumin superfamily	qPH-6, qGY-6c	
Sb06g016230	6	44708205	2.164	Late embryogenesis abundant hydroxyproline-rich glycoprotein	qPH-6, qGY-6c	
Sb06g016260	6	44735351	2.5	Aluminium activated malate transporter	qPH-6, qGY-6c	
Sb06g019470	6	49032854	1.3	Copper transport protein family		qMC1-6a
Sb06g019600	6	49168975	4.279	Cytochrome P450 superfamily protein		qMC1-6a
Sb06g019610	6	49174004	2.452	phosphofructokinase 2		qMC1-6a
Sb06g024400	6	53535970	1.683	NUDIX family, domain containing protein	qMC1-6b	qMC1-6b
Sb06g024590	6	53686490	1.958	tonoplast intrinsic protein	qMC1-6b	qMC1-6b
Sb06g024650	6	53730735	4.367	expansin B2	qMC1-6b	qMC1-6b
Sb06g025220	6	54190773	−2.114	calcium-dependent protein kinase 29	qMC1-6b	qMC1-6b
Sb06g025250	6	54207289	2.782	Prolyl oligopeptidase family protein	qMC1-6b	qMC1-6b
Sb06g025330	6	54262137	2.362	expressed protein	qMC1-6b	qMC1-6b
Sb06g028210	6	57045616	2.252	Terpenoid cyclases/Protein prenyltransferases superfamily protein		qMC2-6
Sb06g028480	6	57260405	2.019	unknown		qMC2-6
Sb06g028760	6	57496434	1.729	Leucine-rich receptor-like protein kinase		qMC2-6
Sb07g022670	7	57311597	2.956	Glutamate decarboxylase	qChl3-7, qBY-7	
Sb07g022800	7	57514740	2.625	aspartyl protease family protein	qChl3-7, qBY-7	
Sb07g023140	7	57977647	4.794	Gibberellin receptor GID1L2	qChl3-7, qBY-7	
Sb07g023220	7	58087984	−3.707	phospholipase A	qChl3-7	
Sb07g023300	7	58178273	2.495	expressed protein	qChl3-7	
Sb07g023770	7	58722654	11.097	rotamase	qChl3-7	
Sb07g024200	7	59189842	−7.492	Ribosomal protein L1p/L10e family	qChl3-7	
Sb07g025190	7	60212195	4.048	MATE efflux family protein	qChl3-7	
Sb07g025240	7	60280418	−2.248	hydroxymethylglutaryl-CoA synthase	qChl3-7	
Sb07g025843	7	60959427	2.544	ethylene-responsive transcription factor ERF114, putative, expressed	qChl1-7	
Sb08g000550	8	482761	−2.111	ferritin-1, chloroplast precursor		qGS-8
Sb08g002590	8	2673615	−2.004	WRKY DNA-binding protein 55		qGS-8
Sb08g002660	8	2779571	2.726	Protease inhibitor/seed storage/LTP		qGS-8
Sb08g003170	8	3513569	3.001	Chalcone and stilbene synthase family		qGS-8
Sb08g003820	8	4423015	−3.008	zinc finger superfamily protein		qGS-8
Sb08g004500	8	5410066	−2.127	fructose-bisphosphate aldolase 2		qGS-8
Sb09g018440	9	46098300	6.288	methyl esterase 3	qAD-9a	qGY-9, qChl2-9a, qBY-9, qChl3-9, qPH-9
Sb09g020000	9	49032969	8.222	inosine-uridine preferring nucleoside hydrolase family protein	qAD-9a	qGY-9, qChl2-9a, qBY-9, qChl3-9, qPH-9
Sb09g020240	9	49471823	3.041	Major facilitator superfamily protein	qAD-9a	qGY-9, qChl2-9a, qBY-9, qChl3-9, qPH-9
Sb09g021016	9	50446536	3.241	ethylene-responsive transcription factor	qAD-9a	qGY-9, qChl2-9a, qBY-9, qChl3-9, qPH-9
Sb09g021250	9	50714173	2.158	alpha/beta-Hydrolases superfamily protein	qAD-9a	qGY-9, qChl2-9a, qBY-9, qChl3-9, qPH-9
Sb09g021490	9	50944384	4.533	Subtilase family protein	qAD-9a	qGY-9, qChl2-9a, qBY-9, qChl3-9, qPH-9
Sb09g021720	9	51194456	−1.869	histone deacetylase 8	qAD-9a	qGY-9, qChl2-9a, qBY-9, qChl3-9, qPH-9
Sb09g022390	9	52044973	8.308	Ribosomal protein	qAD-9a	qGY-9, qChl2-9a, qBY-9, qChl3-9, qPH-9
Sb09g023150	9	52794183	2.46	ribonuclease P family protein	qAD-9a	qGY-9, qChl2-9a, qBY-9, qChl3-9, qPH-9
Sb09g023320	9	52948577	6.593	Major facilitator superfamily protein	qAD-9a	qGY-9, qChl2-9a, qBY-9, qChl3-9, qPH-9
Sb09g024840	9	54319624	−2.168	ferredoxin--nitrite reductase	qAD-9a	qGY-9, qChl2-9a, qBY-9, qChl3-9, qPH-9
Sb09g025530	9	55006797	2.719	O-methyltransferase family protein	qAD-9a	qGY-9, qChl2-9a, qBY-9, qChl3-9, qPH-9
Sb09g025540	9	55018768	2.155	O-methyltransferase family protein	qAD-9a	qGY-9, qChl2-9a, qBY-9, qChl3-9, qPH-9
Sb09g025730	9	55141225	3.406	non-symbiotic hemoglobin 2	qAD-9a	qGY-9, qChl2-9a, qBY-9, qChl3-9, qPH-9
Sb09g025900	9	55284480	−2.24	HSP101 Heat shock protein 101	qAD-9a, qChl2-9b	qGY-9, qChl2-9a, qBY-9, qChl3-9, qPH-9
Sb09g026590	9	55803666	1.86	RING/U-box superfamily protein	qAD-9a, qChl2-9b	qGY-9, qChl2-9a, qBY-9, qChl3-9, qPH-9
Sb09g027380	9	56449825	−2.508	serine/threonine-protein kinase SNT7, chloroplast precursor	qAD-9a, qChl2-9b	qGY-9, qChl2-9a, qBY-9, qChl3-9, qPH-9
Sb09g027470	9	56561299	7.612	Disease resistance protein	qAD-9a, qChl2-9b	qGY-9, qChl2-9a, qBY-9, qChl3-9, qPH-9
Sb09g027590	9	56662520	2.783	seed storage 2S albumin superfamily	qAD-9a, qChl2-9b	qGY-9, qChl2-9a, qBY-9, qChl3-9, qPH-9
Sb09g028890	9	57684814	2.326	Iron-sulfur cluster, SufE/NifU family protein	qAD-9a	qGY-9, qChl2-9a, qBY-9, qChl3-9, qPH-9
Sb09g028960	9	57721281	3.573	ribosomal protein L13	qAD-9a	qGY-9, qChl2-9a, qBY-9, qChl3-9, qPH-9
Sb09g029540	9	58186320	5.903	AMP-dependent synthetase	qAD-9a	qGY-9, qChl2-9a, qBY-9, qChl3-9, qPH-9

Chr, chromosome number; log_2_ ratio; number of folds the gene transcript is differentially expressed in RNA-seq. Log_2_ ratio >0 indicates, positive values indicates gene transcript expressed high in CK60. ns, indicate the transcript is not differentially expressed between CK60 and china17

## Discussion

### Trait variation in the mapping population under different N regimes

The RILs showed transgressive segregation for all the traits measured and in most cases, the mean value of the traits was intermediate between the parental lines, CK60 and China17 (Tables 1 and 2), suggesting a polygenic inheritance of the traits. Transgressive segregation can be caused by both parental lines contributing favorable or unfavorable alleles for a particular trait and is common in inbred populations [55]. In both N conditions, the genetic variance was greater than genotype by environment interaction variance for all the traits (Tables 1 and 2). This finding is in agreement with earlier studies [56]. The more marked contribution of genetic variance to trait determination suggests the opportunity for more robust detection of QTLs that govern nitrogen use efficiency [14]. Here, for both parental lines and RILs marked reductions were observed in mean values for chlorophyll contents measured at three different stages, plant height, biomass and grain yield traits grown under LN compared to NN. In maize, a 38 % reduction in grain yield was observed in plants grown under low-N compared to high-N conditions [14]. This decrease was caused by a significant reduction in kernel number, but has little effect on kernel size. Kernel number is very susceptible to N-stress because ovules are susceptible to abortion soon after fertilization [57], a possible result of limitation in supply of photosynthetic products [58].

### Comparison of QTL regions under contrasting N environments

In this study, a total of 38 QTLs were identified using a SNP based genetic map in the RIL mapping population tested under two different nitrogen levels. However, almost half of these QTLs were detected under one N level, indicating that these traits were controlled by different genes under different N conditions. Major QTLs detected across two normal and two low-N environments were considered as consistent across environments. However, five QTLs for four morphological traits were detected consistently under both N conditions. These included, one QTL each for chlorophyll at maturity, day to anthesis and stover moisture content and two QTLs for head moisture content. For all these QTLs, the CK60 alleles increased chlorophyll content, delayed flowering, and increased stover and head moisture contents under NN and LN. This indicates that these traits shared a similar genetic basis under different N conditions.

### Co-localization of QTLs between traits and associated differentially expressed gene transcripts

Co-localization may suggest pleiotropy whereby a genomic region contains genes that affect a number of traits [59]. In this study, co-localized QTLs affecting different traits were detected on chromosomes 1, 5, 6, 7, and 9 (Fig. 1). For example, the support intervals of ten QTLs explaining 8.1 to 20.3 % of phenotypic variation for eight traits were overlapping in the distal end of chromosome 1. Of the ten QTLs detected, two QTLs are for grain moisture content, one QTL each for test weight, chlorophyll content at anthesis, stover moisture content and grain/stover ratio detected under LN conditions, biomass yield under NN and for days to anthesis detected under NN and LN conditions. An additive effect from CK60 increased days to anthesis (delayed flowering), stover and head moisture content and grain yield. These traits were highly correlated (Table 3) and the correlations resulted in co-localization. Within this co-localized region, QTLs for green leaf area at maturity [60], days to anthesis [60, 61] fresh panicle weight, plant height [59, 62], and panicle architecture [63] were reported earlier. Stay green QTLs and the *Ma3* gene encoding phytochrome B, which is involved in photoperiod sensitivity [64], were also reported in this region.

In this co-localized region containing ten QTLs, RNA-seq detected 19 differentially expressed gene transcripts between CK60 and China17, of which only six DEGs had higher expression in China17 (Table 6). Some of these DEGs including SPX domain-3, Frigida, late embryogenesis abundant protein 1 (LEA) were expressed higher in CK60, and lysine histidine transporter 1 (LHT1) had higher expression in China17. An SPX domain gene-3 was reported to be up-regulated and plays an important role in plant adaptation to phosphate starvation [65]. This region containing a major QTL for days to anthesis, was detected under both N conditions explaining 16 % of phenotypic variation. The CK60 allele contributed to flowering delay by three days. This region contained the flowering time gene transcript, *Frigida,* Which showed more abundant expression in CK60. It was reported earlier that ethylene insensitive 3-Like 1 (EIL-1), key regulator of ethylene biosynthesis, underlies the QTL cluster for days to anthesis, and green leaf area at maturity [60]. However, this gene is not differentially expressed in the root tissues of young seedlings in our RNA-seq analysis (not listed in Table 6). Together, these data suggest that high expression levels of the *Frigida* gene may contribute to the delayed flowering in CK60, but this is not the only gene influencing this phenotype. Similarly another DEG transcript, LEA had two-fold higher expression in CK60 under N-stress condition. Transgenic expression of a barley LEA protein in rice resulted in increased growth rate of transgenic plants than non-transformed plants under stress conditions [66]. Thus, LEA proteins play an important role in protection of plants under stress, a potential tool for genetic improvement towards stress tolerance. In contrast, a DEG transcript encoding high affinity amino acid transporter, lysine histidine transporter (LHT1), was massively expressed in China17 compared CK60 (Table 6). It was reported that being expressed in the root, LHT1 is responsible for uptake of amino acids from soil into root tissue [67], and distributes from roots to shoots through xylem [68] for further metabolism especially under N-stress conditions. The amino acid uptake, and thus nitrogen use efficiency could be higher with increased LHT1 expression under limited inorganic N supply.

A QTL for grain yield is located on distal end of chromosome 1. In this region QTLs for kernel weight [69], maturity [60], number of kernels/panicle and panicle length [70] and panicle architecture [71] were reported earlier. In this region, our RNA seq data detected 20 DEG transcripts including caleosin-related (Ca^+2^ binding) protein, a MADS-box transcription factor, polyamine oxidase 1 were expressed higher in CK60. Gene transcripts for magnesium transporter 6, a heat shock protein (HSP21) and senescence associated protein were more abundant in China17 (Table 6). Polyamines (PAs) and ethylene are endogenous plant growth regulators mediating many physiological processes such as growth, senescence, and responses to environmental stresses [72]. High levels of PAs were reported to be associated with higher kernel set and better seed development in maize [73] and increased grain-filling rates in rice [74]. On chromosome 5, QTLs for biomass yield detected under LN and test weight under NN are co-localized (Fig. 1). For these QTLs, the positive allele from China17 increased biomass yield by 1.0 t.ha^−1^ under LN conditions. In this co-localized region, QTLs for stay green [75, 76], fresh panicle weight and plant height [62] were detected earlier. In this region, RNA seq didn’t detect any significant DEG transcripts between Ck60 and China17.

On chromosome 6, co-localization was observed between major QTLs for plant height and grain yield under LN conditions. For these QTLs, the positive allele from China17 increased plant height by 16.4 cm as well as grain yield. In this region, QTLs for culm height and kernel weight [61], maturity and total dry matter [59], panicle architecture [63] and a major photoperiod sensitivity locus, *Ma1* [77, 78] were reported earlier. Also, a major QTL for plant height, *QPhe-sbi06-1*, conditioned by the *Dw*_*2*_ gene was detected earlier by [60], and showed pleiotropic effects on panicle length, yield, and seed weight [79]. Transcriptome comparison showed that a *Dw2* transcript encoding a multidrug resistance-associated protein 9 homolog showed higher expression levels in CK60, which may be involved in regulating plant height under N-stress in the seedlings (Table 6). In addition, RNA-seq found several differentially abundant gene transcripts in this co-localized region, including auxin response factor 2, seed storage 2S albumin, aluminum activated malate transporter, copper transporter and phosphofructokinase 2, all of which were expressed higher in CK60 and HSP70 was expressed higher in China17. Phosphofructo-2-kinase is the principle enzyme regulating the entry of metabolites into glycolysis [80] through conversion of fructose-6-phosphate to fructose-1,6-bisphosphate. This results in an increase of hexose phosphate, supplying more energy and substrates that are necessary for strong seedling development. It would be of interest to see whether differential expression of these transcripts holds true with the adult tissues and use them in marker assisted selection to regulate the pleotropic regions under LN conditions.

On chromosome 7, QTLs for biomass yield, chlorophyll content at vegetative and maturity were co-localized. For these QTLs, the positive allele from China17 increased biomass yield by 1.0 t.ha^−1^ under LN conditions. In this region, QTLs for fresh total biomass yield and dry total biomass yield was reported by Murray et al. [81]. In this co-localized region, a major plant height gene, *Dw3* (Sb07g0232730), is located. *Dw3* encodes a phosphoglycoprotein auxin efflux carrier orthologous to PGP1 in Arabidopsis [82]. QTL for panicle architecture [61, 69], total biomass yield t.ha^−1^ [81] and plant height [60] were reported earlier. In this region, RNA seq detected 12 DEG’s between CK60 and China 17 (Table 6). Glutamate decarboxylase, gibberellin receptor GID1L2 and ethylene responsive transcription factor ERF114 were expressed higher in CK60 and ribosomal protein L1p/L10e was abundant in China17. Glutamate decarboxylase (GAD1) was reported to be expressed in roots and catalyze the synthesis of γ-aminobutyric acid (GABA) under heat stress, disruption of GAD1 gene prevented accumulation of GABA in roots in response to heat stress [83].

A co-localized region at the distal end of the chromosome 9 contains QTLs for chlorophyll at flowering and days to anthesis across two LN and chlorophyll at maturity, plant height, biomass and grain yield traits across two NN. This clustering of QTLs is supported by the negative correlation observed between the chlorophyll contents at flowering and maturity, morphological and yield-related traits. In this region, alleles from China17 increased plant height, biomass and grain yield but caused negative effects on chlorophyll content at flowering and maturity. QTLs for stay green [76, 84], total seed weight [63], plant height [62], maturity [61, 78] were reported previously in this region. Moreover, a QTL interval for plant height (*Sb-HT9.1*) was fine mapped to ~100 kb region through association mapping [85], *Dw3* and *Sb-HT9.1* were consistently identified as two of the most important plant height loci in crosses between tall and dwarf sorghum [69, 78]. Our RNA-seq data showed that this region contains 28 DEG transcripts including those encoding ferredoxin-nitrite reductase (FNR), chloroplast localized serine/threonine-protein kinase, and a SufE/NifU family protein. FNR gene transcripts were highly expressed in China17 root tissues compared to CK60. In general nitrate is absorbed from soil, reduced to nitrite and then to ammonia by FNR in the plastids of root cells. The ammonia produced is incorporated into amino acids via the glutamine synthetase-glutamate synthase (GS-GOGAT) pathway. This region of chromosome 9 harbors the highly expressed gene encoding NADH-GOGAT and a glutamine-rich protein. However, these genes are not differentially expressed between the root tissues of CK60 and China17 according to RNA-seq data. Further, it would be important to check whether the expression levels of NADH-GOGAT between China17 and CK60 are changed in the shoots because most of the nitrogen assimilation takes place in shoots rather than root tissues. Transgenic over-expression of NADH-GOGAT in rice resulted in an increase in grain weight, indicating that NADH-GOGAT is indeed a key enzyme in nitrogen utilization and grain filling in rice [86]. In wheat, Quraishi et al. [25] validated the NUE QTL on chromosome-3B, and proposed that a GOGAT gene is conserved structurally and functionally at orthologous positions in rice, sorghum and maize genomes and that this gene likely contributes significantly to NUE in wheat and other cereals. It will be of interest to determine if breeding that allows for higher expression of FNR and GOGAT can increase biomass and grain yield by increasing nitrate assimilation and ammonium production.

## Conclusion

QTLs detected for the different agronomic traits in the same genomic regions were consistent with previous QTL mapping studies conducted in diverse genetic and environmental backgrounds in sorghum. RNA-seq analyses detected differential expression of gene transcripts in the pleiotropic QTLs related to nitrogen uptake and metabolism and their expression levels were influenced by the availability of nitrogen. These potential DEG transcripts can possibly be used for improving sorghum performance through marker-assisted selection (MAS) strategies under N-stress conditions by further validation in other mapping populations. The markers and genes reported in this study will have applications in QTL mapping studies, diversity studies, and association mapping studies in sorghum and other members of the Poaceae family collectively aimed at improving nitrogen utilization.

### Availability of supporting data

Supporting data are included as additional files

We deposited the RNA-seq data in Gene Expression Omnibus (http://www.ncbi.nm.nih.gov/geo/query/acc.cgi?acc=GSE54705) and it was mentioned in Gelli et al. 2014, BMC Genomics v15.

## Additional files

Additional file 1:
**Basic parameters showing soil properties at two N levels across years.** (xls 22.0 kb) Additional file 2:
**Genetic distribution of SNPs discovered using genotyping-by-sequencing (GBS) in CK60 x China17 population.** (xlsx 41.1 kb) Additional file 3:
**The list of differentially expressed genes identified between CK60 and China17 using RNA-seq.** (xls 169 kb)

## Abbreviations

RILs: Recombinant inbred lines
QTLs: Quantitative trait loci
SNPs: Single nucleotide polymorphisms
GBS: Genotyping-By-Sequencing
DEG: Differentially expressed gene
NUE: Nitrogen use efficiency
GS: Glutamine synthetase
Ppd: Photoperiod sensitivity
Vrn: Vernalization
Rht: Reduced height
PEPcase: Phosphoenolpyruvate carboxylase
LN: Low Nitrogen
NN: Normal Nitrogen
Chl1: Chlorophyll content at vegetative stage
Chl2: Chlorophyll content at anthesis
Chl3: Chlorophyll content at maturity
PH: Plant height (cm)
AD: Days to anthesis (days)
MC1: Stover moisture content (%)
MC2: Head moisture content (%)
BY: Biomass yield (t.ha^−1^)
GY: Grain yield (t.ha^−1^)
TW: Test weight (g)
GS: Grain/stover ratio (%)
ANOVA: Analysis of variance
IciMapping: Inclusive composite interval mapping
LOD: Logarithm of odds
h^2^: Narrow sense heritability
FDR: False discovery rate
HSP: Heat shock protein
PAs: Polyamines
EIL: 1-ethylene insensitive 3-Like-1
FNR: Ferredoxin-nitrite reductase
GOGAT: Glutamate synthase
